# The Impact of Mechanisms of Action on Adherence and Outcomes in Self-Guided Digital Mental Health Interventions: Protocol for a Randomized Controlled Trial With Mediation Analysis

**DOI:** 10.2196/71238

**Published:** 2025-05-21

**Authors:** Amit Baumel, Idan M Aderka

**Affiliations:** 1 Department of Community Mental Health University of Haifa Haifa Israel; 2 Department of Psychology University of Haifa Haifa Israel

**Keywords:** persuasive design, digital parent training, clinical trial, user engagement, usage, mechanisms of change, mechanisms of action, active ingredients

## Abstract

**Background:**

One of the main recognizable challenges in the digital mental health interventions field is that users adhere to these interventions in their unguided forms poorly. Studies have shown that a persuasive system design focused on encouraging users to make positive behavior changes in their lives can increase user engagement and a program’s efficacy. This design approach can be referred to as therapeutic persuasiveness (TP) and includes a call to action, monitoring, ongoing feedback, and program adaptation based on user state. The goal of this study is to examine the causal impact of TP on program completion and outcomes in unguided digital mental interventions. We aim to examine these questions in digital parent training programs (DPTs) aimed at treating children’s behavior problems.

**Objective:**

This study aims to (1) examine the impact of TP quality on usage, reduction in child behavior problems, and improvement in parenting variables; (2) examine the maintenance of treatment gains over a follow-up period; and (3) examine mediational pathways, including whether adherence to the program (measured by module completion rates) mediates reported changes.

**Methods:**

A randomized controlled trial will be conducted to compare 2 interventions that use the same evidence-based components of established DPTs, but that differ in terms of the quality of TP (standard: DPT-STD; enhanced TP: DPT-TP). We will recruit parents from 160 families with children aged 3-7 years with behavior problems who will be randomized into one of the 2 intervention arms. We will measure child behavior problems and related parenting variables at 5 time points: before (T1), during (T2 and T3), and after the intervention (T4 and T5). Program usage will be passively collected.

**Results:**

The study was funded in October 2023, and enrollment began in September 2024. As of the end of December 2024, 100 participants were enrolled in the study. Analyses are expected to be completed by September 2027.

**Conclusions:**

Identifying conceptual scientific theories that draw a link between active ingredients embedded within a digital intervention function and their outcomes is crucial in advancing our understanding of what influences usage and outcomes.

**Trial Registration:**

ClinicalTrials.gov NCT06514326; https://clinicaltrials.gov/study/NCT06514326

**International Registered Report Identifier (IRRID):**

DERR1-10.2196/71238

## Introduction

### Background

Unguided digital mental health interventions have the potential to increase public access to evidence-based support [1,2]; however, one of the main recognizable challenges in this field is that users poorly adhere to digital programs in their unguided forms (eg, [3-6]). Clearly, a program cannot support its users in reaching beneficial outcomes if it is not being used. Aiming to address this challenge, over the years, more attention has been given to how theorized mechanisms of action reflected in the software’s functions impact program usage, program completion, and the intervention’s effectiveness (eg, [7-9]). Systematic reviews examining the characteristics of digital interventions have suggested that user adherence [10], positive behavior change [11], and program efficacy [12] can be increased by embedding a persuasive system design focused on the incorporation of behavior change techniques. For brevity, we refer to this design approach as therapeutic persuasiveness [13].

Attempts to look under the hood of the digital intervention itself are especially important in light of a randomized controlled trial (RCT) suggesting that adding an unguided digital engagement facilitating intervention before the use of the unguided intervention itself does not increase adherence to the program (defined as number of modules completed) [14]. One of the potential next steps would be to focus on the way the intervention alone should be conceptually designed to increase adherence and efficacy.

### Therapeutic Persuasiveness

Therapeutic persuasiveness addresses how the eHealth program features as a whole are designed to encourage users to make positive changes in their lives. While persuasive system design may be linked to the general use of programs that are not for the benefit of the end user, therapeutic persuasiveness focuses on health-related programs in which users are encouraged to take benevolent actions for their benefit. Following a comprehensive systematic review of published quality criteria of eHealth interventions, we identified several criteria that define a therapeutically persuasive program. The main criteria are a call to action (ie, the provision of triggers to prompt goals and encourage users to complete them), monitoring, ongoing feedback, and program adaptation based on user state and goal achievements [13].

Let us take, for example, the goal of helping parents to increase the number of positive interactions they foster with their children. From the standpoint of a traditional digital program, the main focus would be on providing psychoeducation and suggestions during the use of an eLearning module to increase the occurrence of positive interactions with children. From a therapeutic persuasiveness perspective, however, the intervention designer should also strive to reach parents in their daily environment and make the notion of positive interactions salient in their minds above competing activities. This could be done by triggering parents at the right time to inspire them, monitoring the positive interactions they are engaged with, and providing parents with appropriate acknowledgment and feedback based on their state [15,16].

From the perspective of behavior change theories, the absence of these features, whether delivered through digital components or a human coach, cannot ideally support users’ self-management of desired and undesired behaviors, or their ability to respond to areas of difficulty they may encounter during the therapeutic process (eg, [17,18]). Congruent with these studies, meta-analyses have found that interventions that include self-monitoring components in conjunction with other components (eg, goal setting and feedback on performance) have been significantly more effective [19,20]. Conceptually, programs with a higher quality of therapeutic persuasiveness should therefore be used to a greater extent (resulting in better adherence to the digital program) and deemed more effective.

In correspondence with these ideas, our team examined whether the quality of unguided eHealth interventions could predict the product’s real-world usage, using the Enlight suite of quality ratings (usability, visual design, user engagement, content, therapeutic persuasiveness, therapeutic alliance). Programs’ usage metrics were gathered based on a dataset of anonymized logs from consenting users, simultaneously comparing user traffic between 30 (Microsoft Internet Explorer add-on) and 70 (SimilarWeb Pro panel) different eHealth interventions. The incorporation of therapeutic persuasiveness within the software functions was found to be the most robust and stable predictor of program usage, explaining 11%-42% of the variance in program usage in the regression models [21,22]. These findings are not causal; however, they imply that enhancing the quality of therapeutic persuasiveness as a mechanism of action in unguided interventions may increase the intervention’s acceptability and efficacy. Testing whether this causal relationship exists is the focus of this proposal.

### The Targeted Intervention: Digital Parent Training Program

To examine the proposed causal impact of therapeutic persuasiveness on adherence and efficacy, there is a need to focus on a specific clinical target. This proposal focuses on a digital parent training program (DPT) aimed at treating child behavior problems. This targeted intervention represents a classic case study that can be used to examine the causal impact of therapeutic persuasiveness for several reasons as follows:

- It involves a common public mental health problem. Behavior problems are among the most prevalent types of childhood mental health problems [23,24]. When left untreated, behavior problems impose significant social, emotional, and economic costs, and place a burden on individuals, families, and societies [25,26].
- Parent training aimed at treating child behavior problems is required in an unguided digital form to address the need for increased access to care. While a strong body of evidence supports the efficacy of parent training programs targeting child disruptive behaviors (eg, [27-31]), the provision of evidence-based treatments for children and adolescents is very limited (eg, [32,33]), stressing the need to develop digital forms of care.
- Parents poorly adhere to DPT in its unguided form. DPTs aimed at treating child behavior problems have been introduced over the last decade as a way to increase public access to care [34-36]; however, poor adherence to unguided DPTs has been reported [37,38]. For example, in an RCT of a DPT, Day and Sanders [38] showed that a completely unguided DPT resulted in poor module completion rates, compared with a human-supported condition of the same program (median module completion of 2 and 7, respectively). There is a question as to whether enhancing the therapeutic persuasiveness quality of unguided DPTs could increase program completion rates as well.

### Pilot Study

A pilot study was recently conducted to initially examine the causal impact of therapeutic persuasiveness on user adherence to the program and the program’s efficacy. In this pilot, parents of children with behavior problems were randomly allocated to receive either a standard DPT (DPT-STD) or a DPT designed with enhanced quality of therapeutic persuasiveness (DPT-TP). Both interventions used the same evidence-based content but differed in the quality of their therapeutic persuasiveness. Parents from 88 families who have a child with behavior problems were enrolled in the study. Compared with DPT-STD (n=43), participants allocated to DPT-TP (n=45) used the program significantly more (*P*s<.001; Cohen *d*s=0.91-2.22). In terms of program completion, 68.9% (31/45) of DPT-TP participants completed it compared with 27.9% (12/43) of DPT-STD participants. Significant differences between the interventions were also found in reported improvements in child behavior problems, favoring DPT-TP (*P*s<.05; Cohen *d*s=0.43-0.54) [39].

In a qualitative thematic analysis of semistructured interviews, DPT-TP users identified features embedded exclusively in the enhanced program, such as call-to-action reminders and assessment-based feedback, as important components that helped them in their process of change [40].

### This Study

This study aims to (1) conduct a fully powered RCT design that can provide strong support for the hypotheses; (2) examine the long-term impact of the treatment and the maintenance of treatment gains over a follow-up period, knowledge that is of great importance when aiming to determine whether enhanced mechanisms of action embedded in unguided interventions are maintained over time (or even better in comparison with standard programs); and (3) examine meditational pathways including whether adherence to the program, measured by module completion rates, mediate reported changes.

The study design is a 2-arm parallel-group RCT with repeated measures. It consists of 2 intervention arms for parents of children with behavior problems. We will follow a large sample of families with parents who report having a child with behavior problems, before, during, and after receiving the DPT they are randomly allocated. In particular, this study has the following hypotheses:

1. Adherence and usage: Compared with parents who receive DPT-STD, parents who receive DPT-TP will show better adherence to the digital program (operationally defined as program completion rate) and higher metrics of program usage (number of logins, unique days of use, and total time of use).
2. Efficacy: Compared with parents who receive DPT-STD, parents who receive DPT-TP will report greater improvements in child behavior problems (Eyberg Child Behavior Inventory [ECBI] Intensity), parenting practices (Specific Parenting Practices [SPP], The Parenting Scale [PS], and Alabama Parenting Questionnaire [APQ]) and Parental self-efficacy (The Parenting Tasks Checklist; Me as a Parent [MaaP]), as measured pre-post and pre-follow-up.
3. Mediational pathway: Program completion rate will mediate the impact of therapeutic persuasiveness quality on outcome variables.

In addition, we will explore whether the relationship between program usage and changes in parenting variables and child behavior problems is moderated by program quality (moderated mediation).

## Methods

### Overview

The study and protocol are carried out in accordance with SPIRIT (Standard Protocol Items: Recommendations for Interventional Trials) guidelines (refer to Multimedia Appendix 1 for SPIRIT checklist) and Cochrane Collaboration tool [41,42]. The study was preregistered (ClinicalTrials.gov NCT06514326), and the finalized study protocol number is DPT_08/24. There are no deviations from the original study protocol or trial registry at this time.

### Participants and Recruitment Procedure

#### Eligibility

Parents will be eligible to participate if they (1) have a child aged 3-7 years, (2) report high levels of behavior problems based on the ECBI subscales (ECBI Intensity ≥132; [43]), and (3) have access to a smartphone device with an internet connection. Parents will be excluded if they report that their child is taking medication or is in regular contact with a professional for behavioral or emotional problems, they are currently accessing parenting support elsewhere, or their child has been diagnosed with an intellectual disability or developmental delay. Parents not eligible to participate will be referred to local services.

#### Recruitment Procedure

Parents will be recruited through relevant social media parenting groups, Facebook advertisements, and pamphlets sent to relevant childcare settings. Prospective participants will be directed to the project website, which will offer basic information and a link to a brief eligibility screener consisting of the exclusion criteria and 9 items assessing the parents’ experience of their child’s behavior, based on *Diagnostic and Statistical Manual of Mental Disorders, Fifth Edition* criteria for oppositional defiant disorder (to pass screening parents had to receive an average of 4.22 or above). Screening, interest, and parents’ understanding of the terms of the study will be confirmed by a phone call with a research assistant. Parents will then be directed to a web-based informed consent form, followed by the baseline assessment battery. Once participants have completed baseline assessments and are deemed eligible, they will be randomly assigned to one of the 2 conditions (1:1), stratified for child gender, by an independent researcher who is blind to their assessments and has exclusive access to the assignment sequence. A computer-generated randomization procedure will be used with randomly generated block sizes of 2, 4, and 6. Ineligible participants will be informed about available public mental health care services.

### Measures

The study instruments include self-reported questionnaires that will be assessed at all time points for this study (T1-T5) and data on program usage. Parents will also complete a demographic questionnaire at baseline (T1). Participants will be assessed at 3.3 weeks (T2), 6.6 weeks (T3), and 10 weeks (postintervention; T4), and will complete follow-up assessments at 6 months after the beginning of the intervention (follow-up; T5). The self-report measures will be administered through Qualtrics (Silver Lake).

#### Eyberg Child Behavior Inventory

Child behavior problems will be assessed using the Intensity subscale of the 36-item ECBI [44]. For each item, caregivers rate the intensity of the behavior (1=never to 7=always). The ECBI has proven to be useful in discriminating between problem and nonproblem children for evaluation and in reflecting behavior change problem symptoms following treatment interventions [45,46].

#### The Parenting Scale

Parental disciplinary behaviors in response to their child’s misbehaviors will be assessed using 2 PS subscale scores, Over-reactivity (11 items) and Laxness (10 items), which reflect effective discipline and discipline mistakes on either end, using a 7-point Likert scale. Internal consistency scores (Cronbach α) have been reported (Laxness α=0.83; Over-reactivity α=0.82). These scores have also been found to correlate significantly with observational measures of dysfunctional discipline and child misbehavior [47].

#### Alabama Parenting Questionnaire Positive Parenting Practices

One dimension of parenting practices will be assessed using the APQ Positive Parenting Practices subscale (6 items) [48]. Parents will be asked to rate each item on a scale of 1 (never) to 5 (always) according to how often it typically occurs in their home. The dimension has shown good internal consistency in parents of children, with Cronbach α ranging from α=0.72 to α=0.77 and a test-retest reliability of 0.85 [49,50]. The dimension has also shown criterion validity in predicting externalizing behavioral problems among children [50].

#### The Parenting Tasks Checklist-Self-Efficacy

Task-specific self-efficacy will be assessed using the Setting Self-Efficacy (14 statements) PTC subscale [51]. Item responses are given on a scale of 0 (certain I can’t do it) to 100 (certain I can do it). The subscale has shown excellent internal consistency with a Cronbach α value of 0.91 and has also been found to discriminate between mothers of children with behavior problems who exhibited lower self-efficacy and mothers of a normative sample of children from the community [52].

#### Me as a Parent (Parental Self-Efficacy)

Overall self-efficacy will be assessed using the 4-item Self-Efficacy subscale of MaaP. Sample items include “I have confidence in myself as a parent” and “My parenting skills are effective.” Each item is rated on a Likert scale (1=strongly disagree and 5=strongly agree). The subscale has shown good internal consistency with a Cronbach α value of 0.75 [53].

#### Behaviors and Feelings Survey

The Behaviors and Feelings Survey (BFS)–Internalizing Problems Caregiver version will be used to capture parents’ reports on their child’s emotional problems (eg, anxiety and sadness). It is based on 6 items that are rated on a scale from 0 (not a problem) to 4 (a very big problem). The BFS has demonstrated strong psychometric properties, with evidence supporting its reliability and validity across diverse populations [54].

#### Specific Parenting Practices

Specific parenting practices that are expected to be the direct result of the phases that the program is comprised of will be measured using a scale developed for this study. It includes 12 items. For example, a sample item targeting the practice of “recognizing positive behaviors in your child” asks: “To what extent were you able to express open appreciation and reinforce your child for specific positive behavior demonstrated?” Parents will be asked to rate how often within the past 2 weeks they presented the expected skill on a Likert scale (1=never and 7=always).

#### eHealth Therapeutic Alliance Inventory

The therapeutic alliance in the digital intervention will be assessed at 3 time points (T2-T4) using the eHealth Therapeutic Alliance Inventory (ETAI). It includes 6 items designed to evaluate the strength of the therapeutic relationship in eHealth interventions. Participants rate their agreement with each item on a Likert scale (1=strongly disagree and 7=strongly agree). The inventory has been validated for use in digital health contexts, demonstrating strong reliability and sensitivity to changes in alliance over time [55,56].

#### Quality Assurance

Parents will be invited to respond in writing at postintervention (T4) to the following open-ended questions: “What advantages, if any, did you find in using the program to create a change in the way you react to your child? “Did the program assist you in creating a positive change with your child?” and “What aspects of the program did you appreciate the most?” In addition, perceived usefulness (“The DPT helped me manage my child’s misbehavior or become a better parent”) and satisfaction (“I would recommend the DPT to parents of children with behavior problems”) will be evaluated at postintervention (T4) using a 5-point Likert scale from 1 (I do not agree) to 5 (I agree).

#### Program Completion Rate and Usage

Program usage (eg, login activities, time spent using the program, modules begun and completed) will be automatically documented within the digital platform. Program completion rate is defined as the number of modules the user completes divided by the number of modules the user is expected to complete (7 in DPT-STD and 4-7 in DPT-TP).

### Overview of Interventions

The intervention content includes 7 modules recommended to be completed within 9 weeks. Each module discusses a specific theme: (1) introduction to parent training, (2) positive interactions and quality time, (3) parental emotion regulation, (4) effective routines and clear ground rules, (5) recognizing positive behaviors and ignoring minor negative behaviors, (6) overcoming disobedience, and (7) mindful parenting and conversation between partners.

**Figure 1:**
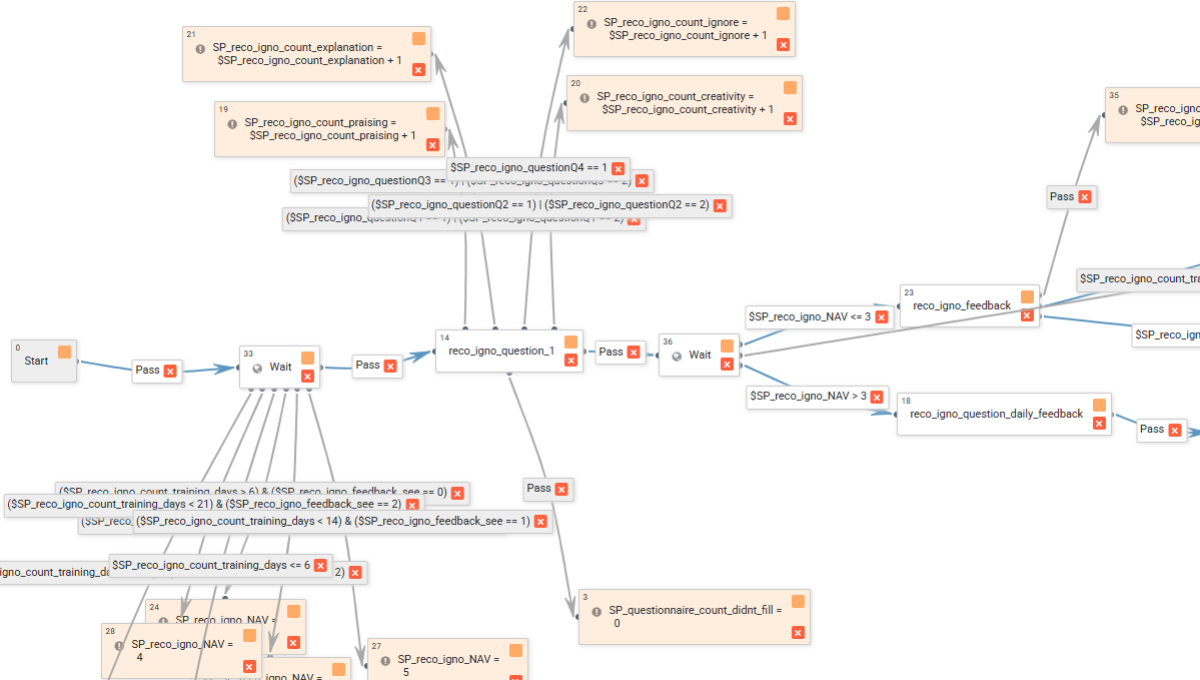
A screenshot with an example of the system’s admin user interface that allows the creation of a logic-driven intervention. Within each arrow, a condition can be created, which determines whether a certain node will be deployed. Blue arrows are between web pages and notifications that the participants view and interact with; gray arrows are back-end processes.

Interventions are delivered through MindTools, an open-source eHealth platform that was originally developed under the name Serafin by Professor Håvar Brendryen from The University of Oslo. The platform was further upgraded by the author (AB) and is available on GitHub [57]. The parenting programs themselves are available to download on Open Science Framework [58]. All content components can be delivered based on logic-driven rules (if-then statements; Figure 1).

#### Standard Digital Parent Training Program

The DPT-STD comprises seven 10- to 25-minute modules, each corresponding to one of the themes mentioned above. Each module’s content includes videos and texts guiding the parent through the training process and interactive features, such as multiple-choice questions with direct feedback. Additional features embedded within the platform include downloadable materials and a “Q&A” section that contains frequently asked questions.

#### Digital Parent Training Program With Enhanced Therapeutic Persuasiveness Quality

The DPT-TP intervention includes all the ingredients of DPT-STD, but with additional features that correspond to the conceptual model of therapeutic persuasiveness, meant to enhance adherence to the program. The program itself is based on predefined decision rules that are either event- or time-based [59], developed in accordance with a user-centered design approach [60]. Each module in the program comprises the learning phase described above, followed by a 1- to 2-week focusing phase.

The focusing phases are designed to help the desired therapeutic activities become salient in the parent’s mind and to help the parent acquire skills in a nonjudgmental manner while avoiding the burden and potential failures that may be associated with the idea of “training.” Accordingly, the program uses the following features.

##### Call to Action

During each focusing phase, parents receive timely triggers (with tips or motivating notes) via SMS text messages that are related to the specific goals and therapeutic activities of the modules they have completed. Triggers draw on accepted paradigms for the tailoring and adaptation of digital triggers (for a review see [59]). For example, in the relevant program phase, motivational messages or tips related to the recognition of positive child behaviors (module 5) are sent 30 minutes before the parents are expected to interact with their child when they come back from work.

##### Monitoring and Ongoing Feedback of User State

Specific practices related to the therapeutic activities or skills that are currently being taught are documented within the system using a brief daily report that includes no more than 7 logic-driven multiple-choice questions and takes less than 2 minutes to complete. These questions ask about parental practices based on a step-by-step script. This is meant to help the parent implement the new way of thinking during their interactions with their child. The program offers nonjudgmental feedback, recognizes desirable achievements based on operant theory, and sends specific messages related to the parent’s current report to help increase motivation for change or reveal new information. For example, across disciplines, positive feedback following positive behavior improves outcomes, whereas negative feedback following failure may reduce motivation and efficacy [61-63].

##### Adaptation to User State

At the end of the first module (introduction), parents will be asked to report about the current state at home, and this will be used to recommend whether to complete the 3 learning modules that are not deemed obligatory: positive interactions, emotion regulation, and effective routines. Parents’ reports on their activities are used to acknowledge their success and to suggest additional actions based on the specific goals reported by parents. Effort related to desired therapeutic activities is adapted based on graded tasks (defined as “load reduction of therapeutic activities” in our therapeutic persuasiveness model [13]). For example, when parents learn how to overcome disobedience (module 6), they will be guided to choose 1 specific behavior problem they would like to focus on first, and then, based on their progress, they will be directed to take additional steps.

#### Therapeutic Persuasiveness Fidelity

Our approach integrates elements such as clear goal relevance, continuous monitoring and feedback, and adaptation to the user’s state. To achieve a satisfying user experience, it is essential that these elements work together effectively. When the users are explicitly required to achieve goals, their goal achievements are expected to be monitored, and it would make sense to also provide them with feedback, and so on. For this reason, the individual components of TP are interrelated and not distinguished in our manipulation. However, using the Enlight quality expert rating scale [13], we evaluated both programs. The DPT-STD design received a TP score of 2.1, which is near the “poor” category, whereas DPT-TP earned a 4.5, placing it between “good” and “very good” scores [39].

### Analytic Plan

#### Preprocessing Steps: Data Audit and Missing Data

The final trial dataset will be accessed by the research coordinators, a statistician, and the principal investigator. Preprocessing will include an examination of data accuracy to identify and correct errors and to ensure that all data are in range. In addition, univariate outliers will be identified (observations that are 3 SDs from the mean in both directions) and will be corrected using winsorizing [64]. Normality (ie, skewness and kurtosis) will be examined for all variables using standard tests (Shapiro-Wilk test and Kolmogorov-Smirnov test). If data significantly differ from a normal distribution according to these tests, we will use transformations depending on the precise deviations from normality (eg, positive skewness, negative kurtosis). Possible transformations will include square root, natural log, inverse, and the Box-Cox transformation [65]. The pattern of missing data will be assessed using Little’s Missing Completely at Random test. Data that are missing completely at random or missing at random will be imputed using multiple imputations with 5 datasets. All preprocessing will be done using SPSS software (IBM Corp).

#### Hypotheses 1-2 Analytic Plan

Hypothesis 1 will be examined using a series of 1-way ANOVAs. Specifically, treatment type will be the independent variable (DPT-STD vs DPT-TP) in all analyses, and program completion rate, number of logins, unique days of use, and total time of use will be the dependent variables. If the treatment conditions significantly differ on important demographic or clinical variables, those variables will be added as covariates to the analysis, creating an analysis of covariance (ANCOVA).

Hypothesis 2 will be examined using two 2-way ANOVAs with treatment type (DPT-STD vs DPT-TP), measurement (pre vs post in the first ANOVA and pre vs follow-up in the second), and their interaction as independent variables. Child behavior problems (ECBI Intensity), parenting practices (SPP, PS scales, and APQ), and parental self-efficacy (PTC and MaaP) will be the dependent variables.

Assumptions of ANOVA will be examined for all analyses and corrected if not met using common procedures. For instance, the assumption of equality of variance will be examined using the Levene test, and if not met, we will use weighted least squares or robust tests such as Welch and Brown-Forsythe.

#### Mediation Analyses (Hypothesis 3 and Exploratory Examination)

**Figure 2:**
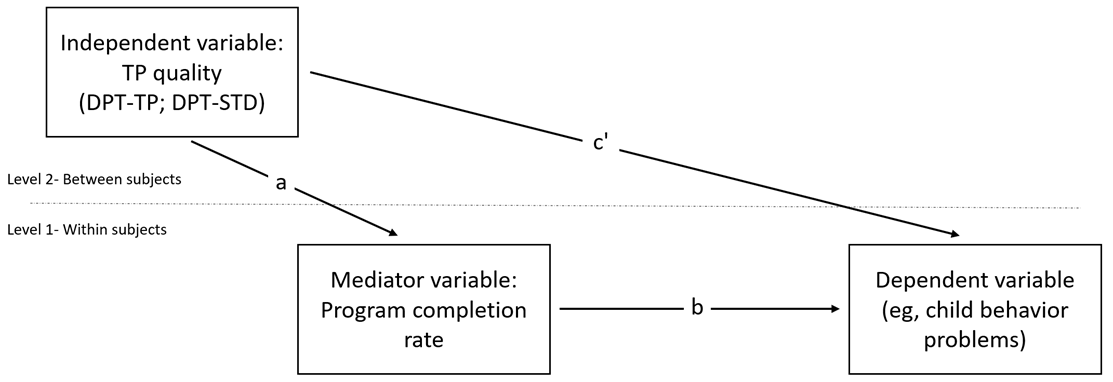
A 2-1-1 mediation model. DPT-STD: standard digital parent training program; DPT-TP: digital parent training program enhanced based on therapeutic persuasiveness quality; TP: therapeutic persuasiveness.

These analyses will be based on the criteria of Zhang et al [66] for mediation in multilevel models. A 2-1-1 mediation model (Figure 2) in which the predictor (TP quality) is a level-2 antecedent variable (between-subjects variable) will be used. The potential mediator (program completion rate) and outcomes (child behaviors and parenting variables) are both level-1 variables (ie, repeated measurements-within-subjects variables). This model tests whether the study manipulation of predictor values influences the values of a level-1 mediator, which then affects a level-1 outcome. Mediation analyses will be conducted using relevant codes for Mplus (Muthén and Muthén) [67,68].

#### Power Estimation

Data emerging from the pilot suggest a large effect size difference in module completion rates and medium effect size differences between the 2 intervention groups in terms of change in ECBI scores and parenting variables post intervention (eg, primary hypothesis Cohen *d*s=0.43; 0.54) [39]. Taking the smaller effect size as an approximation (Cohen *d*=0.43), a total sample size of 80 participants in each of the 2 conditions and a total of 160 participants will provide 0.85 power (α=.05, 1-tailed) to detect significant differences in the primary outcomes (intent-to-treat analysis).

For our mediation analyses, we assume a large effect size difference in program completion rate based on TP quality (the A path), and a small to moderate effect size (correlation) between program completion and change in child behavior problems (the B path). Therefore, a sample size of 160 participants in total is higher than 150 participants, which would be sufficient to examine the relationship between program completion rate (the mediating variable) and child behavior problems (the predicted variable; for a review of sample size estimations for mediation effects, see [69]).

### Ethics Approval

The study received ethical approval from the Institutional Review Board of the University of Haifa (approval number 418/23). All participants provide informed consent before their inclusion in the study (for further details, refer to the Participants and Recruitment Procedure section). Parents will receive a gift card compensation of up to 400 Israeli Shekels (approximately US $113) for their time spent completing assessments at 5 time points, regardless of intervention use. To ensure confidentiality, participant data will be collected using unique identification codes, with the code key securely stored in a separate location. Before data analysis, all data will undergo deidentification to protect participant privacy. No identifying information related to individual participants is present in any images or supplementary materials associated with this paper.

## Results

The study was funded in October 2023, and Table 1 presents the timeline for study completion. As of the end of December 2024, we enrolled 100 participants in the study.

**Table 1:** Timeline for the study completion.

Objective	Beginning	End
Recruitment and eligibility screening of prospective participants	September 2024	June 2026
Conduct baseline assessments (TEST 1)	September 2024	June 2026
Initiate the assigned DPT^a^ condition with participants	September 2024	June 2026
Conduct 3,6, 9-week assessments (TESTS 2-4)	September 2024	September 2026
Conduct 6-month follow-up assessments (TEST 5)	March 2025	December 2026
Analysis and write-up	January 2027	September 2027

^a^DPT: digital parent training program.

## Discussion

Our anticipated principal findings are that, compared with parents in the DPT-STD condition, parents in the DPT-TP condition will demonstrate significantly higher program usage. We also anticipate that program usage will mediate the intervention’s outcomes, meaning that parents in the DPT-TP condition will report greater beneficial changes in their parenting practices and their child’s behaviors at postintervention time points. A key strength of this study is its examination of the process of change across multiple time points, before, during, and after the intervention, providing a unique opportunity to explore mediational pathways. However, a limitation of the study is the potential for beneficial changes occurring between these time points to go undocumented. Nonetheless, as incorporating additional time points introduces a trial bias that is evident in digital interventions [70], we aimed to balance these methodological trade-offs.

Identifying conceptual scientific theories that draw a link between active ingredients embedded within software functions and an unguided intervention’s outcome could be useful in advancing our understanding of what causes and impacts usage and outcomes. Our work has shown that people with limited experience in the eHealth research field can learn how to reliably evaluate the therapeutic persuasiveness quality of a program, achieving high interrater reliability rates for both mobile- and web-based interventions (>0.85) [13]. This finding presents the transparency of this conceptual mechanism of action, which, if deemed relevant, could be useful for scholars worldwide.

From a conceptual perspective, this type of investigation addresses the potential gap between the incorporation of evidence-based content and the incorporation of an evidence-informed therapeutic process (ie, the availability of software functions that impact user interaction with the program and increase its effectiveness). At the clinical level, conducting a fully powered RCT of an unguided DPT for treating child disruptive behaviors in Israel means that, if the program is found effective, the intervention could provide a valuable public health resource for treating child disruptive behaviors throughout Israel, bridging the gap between scientific research and practice [2].

## Appendix

Multimedia Appendix 1 SPIRIT (Standard Protocol Items: Recommendations for Interventional Trials) checklist.

## Abbreviations

ANCOVA: analysis of covariance
DPT: digital parent training program
DPT-STD: standard digital parent training program
DPT-TP: digital parent training program enhanced based on therapeutic persuasiveness quality
RCT: randomized controlled trial
SPIRIT: Standard Protocol Items: Recommendations for Interventional Trials
TP: therapeutic persuasiveness

## Data Availability

The datasets generated or analyzed during this study will not be publicly available prior to the completion of all planned analyses and publications. They will be available from the corresponding author upon reasonable request.
